# Neoadjuvant Chemotherapy with FOLFOX4 Regimen to Treat Advanced Gastric Cancer Improves Survival without Increasing Adverse Events: A Retrospective Cohort Study from a Chinese Center

**DOI:** 10.1155/2014/418694

**Published:** 2014-07-17

**Authors:** Zhen Sun, Rui-Juan Zhu, Gui-Fang Yang, Yan Li

**Affiliations:** ^1^Department of Oncology, Zhongnan Hospital of Wuhan University, Hubei Key Laboratory of Tumor Biological Behaviors & Hubei Cancer Clinical Study Center, No. 169 Donghu Road, Wuchang District, Wuhan 430071, China; ^2^Department of Pathology, Zhongnan Hospital of Wuhan University, No. 169 Donghu Road, Wuchang District, Wuhan 430071, China

## Abstract

*Background/Aim*. To evaluate the clinical efficacy of FOLFOX4 (5-fluomumcil/leucovorin combined and oxaliplatin) neoadjuvant chemotherapy for advanced gastric cancer (AGC). *Patients and Methods*. Fifty-eight AGC patients were enrolled in this retrospective cohort study, 23 in the neoadjuvant group and 35 in the adjuvant group. R0 resection, survival, and adverse events were compared. *Results*. The two groups were well-matched, with no significant differences in R0 resection rate (82.6% versus 82.0%) and number of lymph nodes dissection (16 (0–49) versus 13 (3–40)) between the two groups (*P* > 0.05). The number of lymph node metastases in the neoadjuvant group (3 (0–14)) was significantly fewer than that in the adjuvant group (6 (0–27)) (*P* = 0.04). The neoadjuvant group had significantly better median overall survival (29.0 versus 22.0 months) and 3-year survival rate (73.9% versus 40.0%) than the adjuvant group (*P* = 0.013). The positive expression rate of Ki-67 in the neoadjuvant group (40.0%, 8/20) was lower than that in the adjuvant group (74.2%, 23/31; *P* = 0.015). *Conclusion*. The FOLFOX4 neoadjuvant chemotherapy could improve survival without increasing adverse events in patients with AGC.

## 1. Introduction

In China, over 80% of gastric cancer cases are at clinical stage III or beyond, making them not a local problem but a regional problem. Although local and regional disease control are frequently, but not always, feasible via R0 resection and extended lymphadenectomy, surgical treatment cannot reduce the extraregional recurrence risks that specifically result from hematogenous (visceral) and trans-serosal (peritoneal) progression.

Several approaches to perioperative adjuvant therapy of gastric cancer have shown survival benefits, including postoperative chemoradiation, postoperative systemic chemotherapy, postoperative intraperitoneal chemotherapy, and perioperative systemic chemotherapy [1–4]. Preoperative induction therapy (neoadjuvant chemotherapy) is of particular interest, as it can diminish the disease extend resulting in greater R0 rates; it also provides response assessment with affiliated prognostic implications, as major primary tumor responses have been associated with superior survival. Indeed, in the MAGIC (Medical Research Council Adjuvant Gastric Infusional Chemotherapy) trial, perioperative chemotherapy was shown to increase both the likelihood of curative resection and survival [5].

While preliminary studies have shown the clinical use of neoadjuvant chemotherapy to improve prognoses in advanced gastric cancer (AGC), the optimal regime remains to be determined. Most neoadjuvant chemotherapy regimens were based on fluorouracil (5-FU) and cisplatin. In recent years, the FOLFOX chemotherapy regimen (oxaliplatin, leucovorin, and 5-FU) has been established as a staple of colorectal cancer chemotherapy [6]. This regimen has also achieved remarkable results for gastric cancer in phase II clinical trial [7]. FOLFOX4, one of the FOLFOX chemotherapy regimen, has been investigated by more and more research centers because of its promising clinical efficacy among a variety of cancer types including colorectal cancer, pancreatic cancer, and esophageal cancer [8–10]. Therefore, in order to investigate the potential effect of FOLFOX4 as a neoadjuvant chemotherapy regimen for AGC patients in Chinese population and explore its potential predictive biomarkers, we conducted this retrospective cohort study.

## 2. Patients and Methods

### 2.1. Eligibility

This retrospective analysis included consecutive patients with AGC admitted to the Department of Surgical Oncology, Zhongnan Hospital of Wuhan University from January 1, 2006 to January 30, 2013. Major inclusion criteria were age from 20 to 80 years, WHO performance status 0 to 2, histologically proven gastric carcinoma, no absolution contraindication to surgery and no evidence of distant metastases, as evaluated by CT, chest radiography, and ultrasonography, no history of prior gastric surgery; no previous chemotherapy or radiotherapy, no uncontrolled infectious or cardiac disease; adequate hepatic and renal function, and no previous or other concurrent malignant tumors.

A proportion of these patients, who were diagnosed as resectable T3 or T4 cancers with or without nodal involvement (as determined by CT), underwent preoperative chemotherapy with a combination regimen (FOLFOX4) consisting of oxaliplatin, leucovorin, and 5-fluorouracil (5-FU) followed by curative surgical resection and postoperative chemotherapy mainly FOLFOX (neoadjuvant group). The adjuvant group selection was divided into two steps. During the first step, patients with T3/T4 gastric cancer treated with surgery first and followed by adjuvant FOLFOX chemotherapy were selected by surgeon investigators from their records. During the second step, the principal investigator double-checked the medical records of the potentially eligible patients by recontacting the investigators to ensure that the eligibility criteria had been applied homogeneously. During double-checking, the investigator was blinded to the detailed characteristics of the patients in the neoadjuvant group. We adopted a 1 : 1.5 ratio of exposed: unexposed proportion. This study was approved by the institutional review board of the hospital and informed consent was obtained from each patient.

### 2.2. Neoadjuvant Chemotherapy and Response Assessment

In the neoadjuvant group, patients received 2 to 6 cycles of preoperative FOLFOX4 regiments consisting of oxaliplatin at 85 mg/m^2^ on day 1 and leucovorin at 200 mg/m^2^ administered intravenously for 2 hours followed by 5-FU at 400 mg/m^2^ as a bolus followed infusion 5-FU at 600 mg/m^2^ by continuous infusion for 22 hours on days 1 and 2. Antiemetics and granulocyte-colony stimulating factor (G-CSF) were prescribed when required. Before each cycle of chemotherapy, the tumor markers including CEA, CA125, and CA199 and a complete blood count were obtained and blood urea nitrogen, electrolyte, serum creatinine level, and liver function were determined. Adverse events graded according to the National Cancer Institute Common Toxicity Criteria version 4.0 [11] were recorded during hospitalization. The regimen was repeated every 2-3 weeks.

Assessment of response to neoadjuvant therapy was based on reduction of primary tumor size measured by CT scan (RECIST evaluation criteria) [12] and the decreases of the tumor markers (TMs) measured before and after the neoadjuvant chemotherapy using the criteria as follows [13]: complete clinical response (CR), normalization of all measured TMs; partial response (PR), a 50% or greater decrease in the values of TMs with initially elevated values; stable disease (SD), changes in TM values of less than 50% in two of the three TMs listed above; progressive disease (PD), an increase of 50% or more in the value of at least one TM. According to the criteria above, if a PR or a CR happened, two further courses of chemotherapy were given prior to surgery. In all other cases, the patients became eligible for surgery after the second course of chemotherapy.

### 2.3. Surgery and Pathological Evaluation

Resection of the gastric tumor was performed within 1 week after hospital admission for patients in the adjuvant group and 3 to 4 weeks after completion of chemotherapy in the neoadjuvant chemotherapy group. After laparotomy, the extent of dissection and whether the surgical procedure was likely to be curative (R0) were decided; R1 indicated microscopic evidence of tumor cells at the margin of the resection, whereas R2 indicated macroscopic evidence of tumors beyond the margin of the resection. For patients with R0 resections, a D2 lymphadenectomy was performed according to the Japanese gastric cancer treatment guidelines 2010 (ver.3) [14]. All resected tissues were examined according to a standardized histopathological protocol, with evaluation of the TNM stage according to the 7th edition of American Joint Committee on Cancer (AJCC) TNM staging system for gastric cancer. Histopathological response is assessed by two independent and blinded pathologists using the criteria described by the Japanese Research Society for Gastric Cancer [15]. Grade 0 represents no response (no necrosis or cellular or structural changes within the tumor); grade 1a represents the presence of necrosis or disappearance of the tumor in <1/3 of the entire tumor or only cellular structural changes and grade 1b is the presence of necrosis or disappearance in more than 1/3 of the entire cancer. Grade 2 indicates moderate change with necrosis or disappearance of the tumor in more than 2/3 of the entire lesion, but viable cells still remain and grade 3 indicates marked change with necrosis or disappearance of the tumor, or replacement by fibrosis in the entire lesion with no viable cells remaining. Following surgery, the patients in both groups received 1–6 cycles of postoperative adjuvant treatment mainly using the same FOLFOX regime.

### 2.4. Follow-Up

All patients were followed until death or until the date of last followup as of 30 January, 2013. The presence of a relapse was determined by appropriate imaging studies. Overall survival (OS) was defined as the interval between commencement of treatment and death. Progression-free survival (PFS) was measured from commencement of treatment to occurrence of an event—relapse or death—whichever came first. Data on patients who were event-free were censored on the date of last followup.

### 2.5. Immunohistochemistry (IHC)

Immunolocalization of Ki-67, c-erbB-2, CD34, and MMP9 (matrix metalloproteinase-9) was performed using avidin-biotin-peroxidase complex (ABC) method. Briefly, tissue slides were first deparaffinized in xylene, ethanol, and water; then, the slides were pretreated in 0.01 M citrate buffer (pH 6.0) and heated in a microwave oven (98°C) for 15 min. For staining, endogenous peroxidase activity was blocked by immersing in 3% hydrogen peroxide/methanol buffer for 20 min at room temperature. After washing in PBS, the slides were incubated with the primary antibodies for Ki-67 (MAB-0129, Maixin Biotechnology, China, working solution), c-erbB-2 (RMA-0555, Maixin Biotechnology, China, working solution), CD34 (MAB-0034, Maixin Biotechnology, China, working solution), and MMP9 (sc13595, Santa Cruz, USA, dilution 1/300) overnight at 4°C. Then the sections were washed with PBS and incubated with polymerase auxiliaries for 20 min. After washing in PBS, the sections were incubated with the biotinylated secondary antibody for 60 min at room temperature, and finally DAB (diaminobenzidine) was visualized. As a negative control, primary antibody was replaced with Tris-buffered saline on sections that were proven to be positive for Ki-67, c-erbB-2, CD34, and MMP9 in preliminary experiments.

### 2.6. Evaluation of Immune Staining

All slides were independently observed by two investigators who were blinded to the clinicopathological characteristics of patients. A consensus score was agreed for each slide by the investigators.

The percentage of positively stained cells was calculated after 100 cells were counted in more than 5 high-power (×400) fields. The following definitions were made: Ki-67: more than 10% positive staining in nuclei was defined as positive staining; c-erbB-2 and MMP9: more than 10% positive staining in cytoplasm was defined as positive. For MVD, assessment involved the initial identification of highly vascular areas by scanning the entire section at low magnification (×100), defined as areas having the highest density of CD34-positive cells; microvessels were counted (×400 field) by light microscopy in each of the five most vascularized areas, and necrotic and ulcerated areas were avoided [16].

### 2.7. Statistical Analysis

All data analyses were performed using the SPSS statistical software program, version 19.0 (SPSS Inc., Chicago, IL, USA) for Windows. Categorical data were compared using chi-square tests. Tumor markers, number of nodes harvested, and metastatic lymph nodes were compared using nonparametric Wilcoxon test. The Kaplan-Meier analysis was used to estimate survival rates and analyzed by Log rank test. The Cox proportional-hazards model was used to calculate the hazard ratios. Univariate analysis of associations was determined using Spearman's correlation analysis. *P* < 0.05 was considered as statistically significant.

## 3. Results

### 3.1. Characteristics of the Patients

Between January, 2006, and January, 2012, 58 patients were recruited, 23 in the neoadjuvant group and 35 in adjuvant group. As shown in Table 1, major clinicopathological characteristics were balanced (Table 1).

### 3.2. Evaluation of Responses to Neoadjuvant Chemotherapy

Serum levels of CEA, CA125, and CA199 were significantly decreased after neoadjuvant chemotherapy (*P* < 0.05) (Table 2). When combined CEA, CA125, and CA199 as an indicator to evaluate the efficacy of neoadjuvant chemotherapy, there were 7 patients (30.4%) having CR and PR each and 7 patients (30.4%) had SD and 2 patients (6.1%) had PD. The TMs response rate (CR + PR) was 60.9% (14/23). The clinical tumor responses were evaluated using contrast enhanced CT scans according to the RECIST criteria; the overall major response rate was 43.5% (10/23) where no patient had CR and 10 patients (43.5%) had PR, 9 patients (39.1%) had SD, and 4 patients (17.4%) had PD.

### 3.3. Operation Data

The R0 resections rate was 82.6% (19/23) in the neoadjuvant group and 80.0% (28/35) in the adjuvant group (*P* = 0.807). No perioperative mortality was observed. Postoperative complications were comparable between the two groups (Table 3).

### 3.4. Pathological Findings

According to the Japanese Gastric Cancer Research pathological evaluation standard of chemotherapy effects, no patient had grade 3 pathological response, 8 (34.8%) had grade 2 response, 10 (43.5%) had grade 1b response, 3 (13.0%) had grade 1a response, and 2 (8.7%) had grade 0 response. The median number of dissected lymph nodes was similar in both arms, (16 (0–49) in neoadjuvant group versus 13 (3–40) in adjuvant group). The median number of positive lymph nodes was 3 (0–14) in the neoadjuvant group versus 6 (0–27) in the adjuvant group (*P* = 0.040). Also, there was a significant trend towards less advanced nodal disease in the neoadjuvant group than in the adjuvant group (*P* = 0.034) (Table 3).

### 3.5. FOLFOX4 Chemotherapy Toxicity Evaluation

The most common toxicities were hematological and gastrointestinal toxicity. In all cases, the toxic effects resolved after treatment completed and no treatment was terminated because of toxicity. There was no clinically significant difference in the incidence of the toxic effects associated with the chemotherapy between two groups (Table 4).

### 3.6. Survival

**Figure 1:**
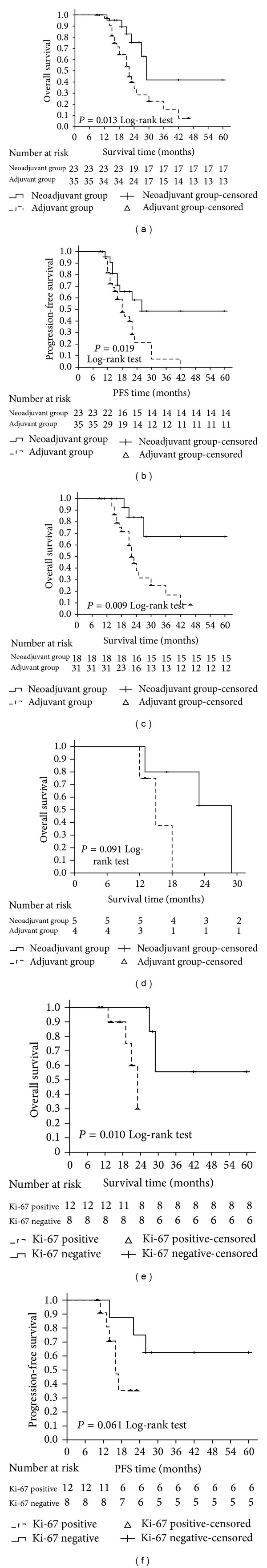
Kaplan-Meier analysis of the neoadjuvant group and adjuvant group. OS (overall survival) and PFS (progression-free survival) between the two groups ((a), (b)); OS between the two groups without metastasis (c); OS between the two groups with metastasis (d); ((e), (f)) Kaplan-Meier analysis of the Ki-67 positive and negative patients in the neoadjuvant group. Ki-67 negative patients had greater OS benefits than Ki-67 positive patients in the neoadjuvant group (e). There was also a trend towards better PFS benefits in Ki-67 negative patients than positive patients, although the difference did not reach statistical significance (f).

The median followup was 26.0 months (10.0–61.0 months) in the neoadjuvant group and 31.0 months (15.0–72.0 months) in the adjuvant group. The median OS for patients in the neoadjuvant and adjuvant groups were 29.0 months (95% CI, 25.3–32.7 months) and 22.0 months (95% CI, 18.2–25.8 months), respectively. The median PFS were 26.0 months (95% CI, not reached) and 18.0 months (95% CI, 14.4–21.6) respectively. The 3-year OS was 73.9% (95% CI, 54.6%–93.2%) in the neoadjuvant group and 40% (95% CI, 30.1%–49.9%) in the adjuvant group (*P* = 0.013) (Figure 1(a)). The overall 3-year PFS was 60.9% (95% CI, 47.7%–74.1%) in the neoadjuvant group compared with 34.3% (95% CI, 26.2%–42.4%) in the adjuvant group (*P* = 0.019) (Figure 1(b)).

Multivariate Cox proportional hazards analysis indicated that neoadjuvant chemotherapy (hazards ratio [HR] = 0.202 (95% CI, 0.072–0.570), *P* = 0.003) and distant metastasis (HR = 5.388 (95% CI: 1.856–15.643), *P* = 0.002) were two independent prognostic factors for OS after excluding other confounding factors. Therefore, we performed subgroup analysis to determine if there was a differential survival benefit of neoadjuvant chemotherapy in the patients with distant metastasis. In patients without distant metastasis who underwent neoadjuvant therapy versus those who did not, the 3-year OS was 83.3% versus 41.9% (*P* = 0.009) (Figure 1(c)), whereas in patients with distant matastasis, there was no clear evidence of treatment effect on OS between those who received neoadjuvant treatment and those who did not (*P* = 0.091). However, due to the small number of patients with M1 stage, the statistical power was limited (Figure 1(d)).

### 3.7. Immunohistochemical Analyses and Clinicopathological Correlations

A total of 20 specimens were suitable for immunohistochemical analyses in the neoadjuvant group and 31 in the adjuvant group.

**Figure 2:**
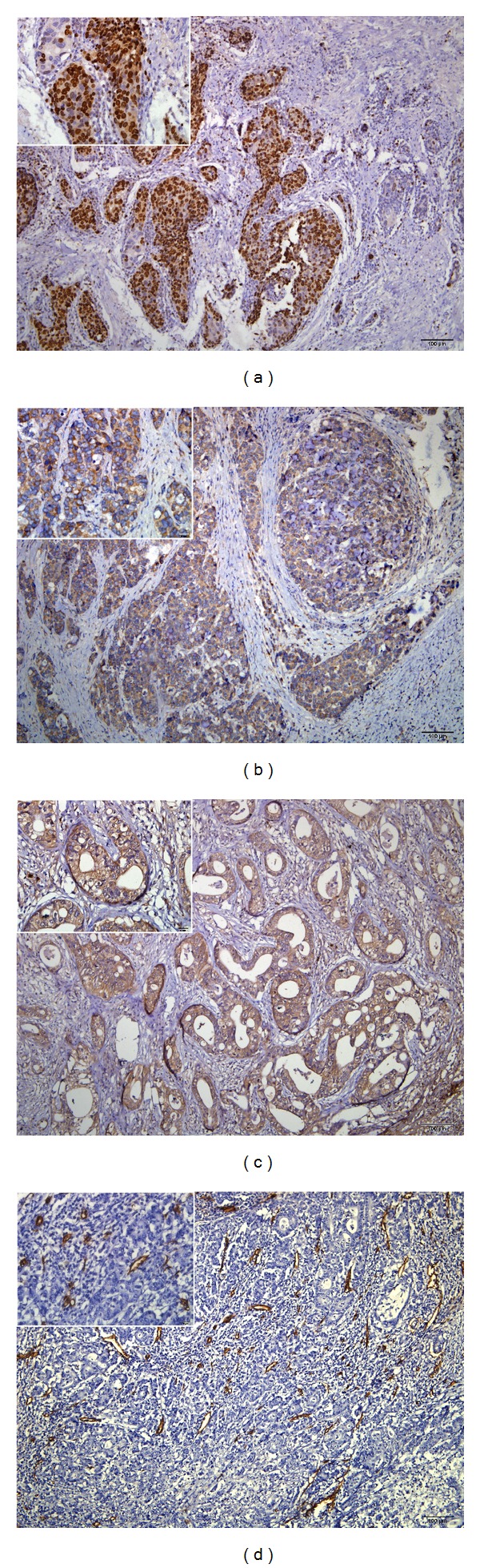
Immunohistochemical staining of Ki-67 ((a) brown stain in nuclei), c-erbB-2 ((b) brown stain in membrane and cytoplasm), MMP-9 ((c) brown stain in membrane and cytoplasm) and microvessels. (d) Original magnification 100x; insets 400x. All tissues were adenocarcinoma of GC.

Ki-67 positive rate in the neoadjuvant group (60.0%, 12/20) was slightly lower than that in the adjuvant group (74.2%, 23/31; *P* = 0.286) (Figure 2). Ki-67 positive tumors had a negative correlation with histopathological response to neoadjuvant chemotherapy as evidenced by a response rate of 2 or 3 (*P* = 0.007). There were no significant associations between Ki-67 expression and other variables such as invasion depth, nodal involvement, and TNM stage. The median OS in Ki-67 positive patients was 21.1 versus 45.9 months in Ki-67 negative patients (*P* = 0.010; Figure 1(e)). The median PFS in Ki-67 positive patients was 17.6 versus 45.3 months in Ki-67 negative patients (*P* = 0.061; Figure 1(f)).

The positive expression rate of c-erbB-2 in the neoadjuvant group (10.0%, 2/20) was similar to that of the adjuvant group (16.1%, 5/31; *P* = 0.690). There was no correlation between c-erbB-2 expression and pathological response or other clinicopathological variables.

The positive expression rate of MMP-9 in the neoadjuvant group (55.0%, 11/20) was similar to that in the adjuvant group (67.7%, 21/31; *P* = 0.358). MMP-9 expression had no correlation with pathological response or other clinicopathological variables.

The median MVD value was 19. When this median value was determined as the cut-off point; the high MVD rate in the neoadjuvant group (45.0%, 9/20) was similar to that in the adjuvant group (51.6%, 16/31; *P* = 0.645). MVD had no correlation with pathological response or other clinicopathological variables.

## 4. Discussion

The prognosis of AGC is poor despite seemingly curative resection [17]. Several studies have demonstrated the benefit of neoadjuvant chemotherapy for resectable locally AGC [5, 18, 19]. The results of the first stage III clinical randomized trial (MAGIC) of neoadjuvant chemotherapy of gastric cancer indicated that neoadjuvant chemotherapy could significantly lower tumor stage, increase the rate of R0 resection (79% versus 69%), and improve 5-year survival (36% versus 23%). The MAGIC trial provides a high-level clinical evidence for the application of the neoadjuvant chemotherapy in resectable locally AGC. However, there is still a lack of definitive evidence for specific neoadjuvant chemotherapy regime. Hence, the platinum-based multidrug combinational regimen was recommended in the 2011 version of the NCCN gastric cancer practice guidelines.

The use of oxaliplatin, 5-FU, and leucovorin (FOLFOX) in this setting has been under investigation, which prompted this study to investigate the effectiveness of neoadjuvant FOLFOX in the treatment of gastric cancer. Since 2001, the FOLFOX regimen had become one of the most common treatments for AGC. Al-Batran et al. [20] and Luo et al. [21] used FOLFOX6, while de Vita et al. [22] used FOLFOX4 as neoadjuvant chemotherapy for gastric cancer, the clinical effective rate was 43%, 41%, and 38%, respectively. Li et al. [23] treated 36 patients with advanced (T3 or T4 TNM staging) gastric cancer who received neoadjuvant FOLFOX; complete and partial responses were observed in 2 (6%) and 21 (64%) patients; moreover, the chemotherapy was well tolerated by all patients. Zhang et al. [24] reported modified FOLFOX7 showing a clinical effective rate of 50%. In our study, the clinical response rate observed was 43.5% (0% CR and 43.5% PR), which is similar to the results reported above.

Clinical response rate is an important indicator of judging the efficacy of chemotherapy; however, a variety of adverse events caused by chemotherapy should also be observed. Pre-operative ECF has been previously reported to achieve response rates of 49% to 56% [5, 25, 26]. However, perioperative ECF is also associated with grade 3/4 neutropenia in 23.8%, and grade 3/4 nonhematologic toxicities in 2–6% each in nausea, vomiting, diarrhea, stomatitis, neurological effects, and skin changes [5]. Compared with ECF regimen in the MAGIC trial, the FOLFOX4 regimen seems well tolerated and safe with few patients experiencing grade 3/4 toxicity. In Al-Batran and de Vita Studies [20, 22], the FOLFOX regimen did not lead grade 3/4 neurological toxicity or neutropenia, and patients with grade 1/2 neutropenia or gastrointestinal reactions recovered after symptomatic treatment. In our study, most toxicities were grade 1/2, and no patients withdrew from chemotherapy because of toxic side effects and there were no chemotherapy related deaths.

The survival benefits of neoadjuvant chemotherapy for AGC warrant further discussion. In this study, the three-year OS in the neoadjuvant group was 73.9% (95% CI, 54.6%–93.2%) as opposed to 40.0% (95% CI, 30.1%–49.9%) in the adjuvant group. This finding is consistent with two recent large scale randomized trials. MAGIC and FNLCC trials [5, 19] have reported that the five-year OS in neoadjuvant chemotherapy group were 36% and 38% which were significantly higher than that in the adjuvant chemotherapy group. However, in Wang and Nio trials [27, 28], the five-year OS in neoadjuvant chemotherapy group were 40% and 72% which had no significant advantage compared with surgery alone group. The first two trials are the European multi-center clinical study, and their large sample size and strict randomization, helped obtain a powerful evidence of neoadjuvant chemotherapy for AGC. But their different D2 gastrectomy practice compared with Asian procedures may limit its applicability. In comparison, the latter two Asian multicenter clinical studies may have a large range of radical surgery, but their small sample size and lack of strict randomization limited their statistical power. Therefore, the standard surgical approach, strict randomization, and large samples should be three important factors to judge the survival benefit of neoadjuvant chemotherapy in the future clinical trials.

We also investigated the potential biomarkers that may further evaluate the efficacy of the neoadjuvant chemotherapy. We focused on the expression of Ki-67, c-erbB-2, MMP-9, and CD34 in postoperation samples in order to investigate their reactions towards neoadjuvant chemotherapy and analyze their prognostic and predictive potential in the patients treated with FOLFOX4 neoadjuvant regimen. A lower Ki-67 positive expression rate was observed in the neoadjuvant group implying that FOLFOX4 chemotherapy may have influenced the tumor cell proliferation in gastric cancer. We also show that nuclear expression of Ki-67 is negatively associated with histopathological response to neoadjuvant chemotherapy implying that Ki-67 could be a promising predictive marker. However, the pretreatment Ki-67 level still needs to be investigated in order to further assess the role of Ki-67 as a predictive factor of responsiveness to chemotherapy or other biological therapies. Moreover, we have also shown that nuclear Ki-67 expression correlates with poor OS (*P* = 0.010) in neoadjuvant group which can be considered that Ki-67 is likely to have a prognostic significance in these tumors. Recently, Ellis et al. [29] have reported that Ki-67 index after neoadjuvant hormonal therapy is a significant prognostic factor in breast cancer probably because it represents the antitumor effect of hormonal therapy. Similarly, Lee et al. [30] and Jones et al. [31] have investigated the prognostic value of Ki-67 index after neoadjuvant chemotherapy and have reported that Ki-67 index after neoadjuvant chemotherapy is a significant prognostic factor. Our results are consistent with the above findings. In addition, to our knowledge we have provided the first evidence of Ki-67 index as a prognostic factor in gastric cancer patients with FOLFOX4 neoadjuvant regimen. Hence, postneoadjuvant chemotherapy Ki-67 level could be a prognostic factor in gastric cancer patients and might be clinically useful in the prediction of patient prognosis and the decision making as to the indication of a further adjuvant therapy. The expression of c-erbB-2, MMP-9, and CD34 did not differ between two groups and did not correlate with clinicopathological factors in patients with neoadjuvant group. We can infer that the FOLFOX4 regiment may have an impact on the tumor cell proliferation, but its influence towards the tumor microenvironment features after neoadjuvant chemotherapy needs to be further investigated by prospective clinical studies with large scales.

Our study is limited by the retrospective design and small sample size. However, we have provided evidence that the FOLFOX4 regimen as neoadjuvant chemotherapy could reduce lymph node metastasis and improve survival without increasing adverse events in patients with AGC. We also have provided evidence that postneoadjuvant chemotherapy Ki-67 level could be a promising predictive biomarker in patients with advanced gastric cancer who receive FOLFOX4 regimen. To further validate FOLFOX4 regimen that is likely to have important clinical implications for patients receiving neoadjuvant chemotherapy for AGC, randomized controlled trial with larger sample size will be done in the near future in our department based on the preliminary but promising results in this study.

## Figures and Tables

**Table 1:** Patient demographics and tumor characteristics.

Items	Neoadjuvant group (*n* = 23)	Adjuvant group (*n* = 35)	*P* value∗
Median age (yr) (range)	58 (34–79)	57 (31–80)	0.760
Gender: *n* (%)			
Male	15 (65.2)	22 (62.9)	0.855
Female	8 (34.8)	13 (37.1)	
Anatomic location: *n* (%)			
Proximal	4 (17.4)	3 (8.6)	0.490
Body	7 (30.4)	9 (25.7)	
Distal	12 (52.2)	23 (65.7)	
Tumor grade: *n* (%)			
Well/moderately differentiated	6 (26.1)	8 (22.8)	0.701
Poorly differentiated	11 (47.8)	20 (57.1)	
Mucinous/signet ring cell cancer	4 (17.4)	6 (17.2)	
Others	2 (8.7)	1 (2.9)	
Pretreatment clinical T-stage (CT): *n* (%)			
Stage 3	17 (73.9)	28 (80.0)	0.587
Stage 4	6 (26.1)	7 (20.0)	

*Comparisons for categorical variables performed using chi-square test.

**Table 2:** Tumor marker responses after the neoadjuvant chemotherapy.

Items	Preneoadjuvant chemotherapy (*n* = 23)	Postneoadjuvant chemotherapy (*n* = 23)	*P* value∗
CEA median (range)	0.84 (0.05–14.40)	0.70 (0.17–13.90)	0.030
CA125 median (range)	0.77 (0.25–14.26)	0.45 (0.19–6.28)	0.003
CA199 median (range)	0.24 (0.05–59.46)	0.20 (0.00–25.31)	0.005

*The tumor markers were expressed as folds over upper normal limit. *P* values for tumor markers were calculated using Wilcoxon test.

CEA: carcinoembryonic antigen; CA125: cancer antigen 125; CA199: carbohydrate antigen 199.

**Table 3:** Surgical and pathological results.

Items	Neoadjuvant group (*n* = 23)	Adjuvant group (*n* = 35)	*P* value∗
Margins: *n* (%)			
R0	19 (82.6)	28 (80.0)	0.807
R1/2	4 (17.4)	7 (20.0)	
Postoperative complication: *n* (%)			
Anastomotic leak	1 (4.3)	0 (0)	0.507
Respiratory infection	2 (8.7)	3 (8.6)	
Postoperative hemorrhage	1 (4.3)	1 (2.9)	
Postoperative bowel obstruction	0 (0)	2 (5.7)	
Pathological staging			
T-stage: *n* (%)			
T1	1 (4.3)	2 (5.7)	0.335
T2	5 (21.7)	2 (5.7)	
T3	4 (21.7)	8 (22.9)	
T4	13 (52.1)	23 (65.7)	
N-stage: *n* (%)			
N0	8 (34.8)	2 (5.7)	0.034
N1	3 (13.0)	9 (25.7)	
N2	4 (17.4)	10 (28.6)	
N3	8 (34.8)	14 (40.0)	
Number of nodes harvested: *n*	16 (0–49)	13 (3–40)	0.886
Number of metastatic lymph nodes: *n*	3 (0–14)	6 (0–27)	0.040

*Comparisons for categorical variables performed using chi-square test. *P* values for number of nodes harvested and metastatic lymph nodes were calculated by Wilcoxon test.

**Table 4:** Adverse effects associated with preoperative and postoperative chemotherapy.

Adverse events	Neoadjuvant group (*n* = 23)	Adjuvant group (*n* = 35)	*P* value∗
Neutropenia: *n* (%)			
Grades 1-2	6 (26.1)	7 (20.0)	NS
Grades 3-4	3 (13.0)	3 (8.8)	
Nausea and vomiting: *n* (%)			
Grades 1-2	4 (17.4)	8 (22.9)	NS
Grades 3-4	1 (4.3)	2 (5.7)	
Liver toxicity: *n* (%)			
Grades 1-2	1 (4.3)	2 (5.7)	NS
Grades 3-4	0 (0)	0 (0)	
Stomatitis: *n* (%)			
Grades 1-2	2 (8.7)	1 (2.9)	NS
Grades 3-4	0 (0)	0 (0)	
Diarrhea: *n* (%)			
Grades 1-2	2 (8.7)	2 (5.7)	NS
Grades 3-4	0 (0)	2 (5.7)	
Neurologic effects: *n* (%)			
Grades 1-2	4 (17.4)	2 (5.7)	NS
Grades 3-4	0 (0)	0 (0)	
Skin effects: *n* (%)			
Grades 1-2	1 (4.3)	1 (2.9)	NS
Grades 3-4	0 (0)	0 (0)	

*Comparisons for categorical variables performed using chi-square test. NS: no significance.
